# The social life of cyanobacteria

**DOI:** 10.7554/eLife.70327

**Published:** 2021-06-16

**Authors:** Conrad W Mullineaux, Annegret Wilde

**Affiliations:** 1 School of Biological and Chemical Sciences, Queen Mary University of LondonLondonUnited Kingdom; 2 Institute of Biology III, University of FreiburgFreiburgGermany

**Keywords:** *Synechocystis sp*. PCC 6803, cyanobacteria, exopolysaccharide, bloom, Other

## Abstract

The cyanobacterium *Synechocystis* secretes a specific sulphated polysaccharide to form floating cell aggregates.

**Related research article** Maeda K, Okuda Y, Enomoto G, Watanabe S, Ikeuchi M. 2021. Biosynthesis of a sulfated exopolysaccharide, synechan and bloom formation in the model cyanobacterium *Synechocystis sp*. strain PCC 6803. *eLife* **10**:e66538. doi: 10.7554/eLife.66538

Cyanobacteria are ancient and extremely versatile organisms that can be found in nearly every ecosystem on Earth, in particular lakes, rivers and oceans. Like plants and algae, they produce oxygen and use sunlight as an energy source.

Some cyanobacteria – even single-celled ones – show striking collective behaviours and form colonies (or ‘blooms’) that can float on water and have important ecological roles. For instance, billions of years ago, communities of marine Paleoproterozoic cyanobacteria could have helped create the biosphere as we know it by burying carbon compounds and allowing the initial build-up of oxygen in the atmosphere (Kamennaya et al., 2018). On the other hand, toxic cyanobacterial blooms are an increasingly issue for society, as their toxins can be harmful to animals (Huisman et al., 2018). Extreme blooms can also deplete water of oxygen and reduce sunlight and visibility, thereby compromising the feeding and mating behavior of light-reliant species.

It has been unclear why and how cyanobacteria form communities. Aggregation must divert resources away from the core business of making more cyanobacteria, as it generally involves the production of copious quantities of extracellular material. In addition, cells in the centre of dense aggregates can also suffer from both shading and shortage of nutrients (Conradi et al., 2019; Enomoto and Ikeuchi, 2020). So, what advantage does this communal life bring for cyanobacteria?

Now, in eLife, Masahiko Ikeuchi of the University of Tokyo and colleagues – including Kaisei Maeda as first author – report new insights into how cyanobacteria form blooms (Maeda et al., 2021). Using the widely studied cyanobacterium *Synechocystis*, they identified a set of genes that regulate the production and export of sulphated polysaccharides, chains of sugar molecules modified with sulphate groups that can often be found in marine algae and animal tissue. Many bacteria generate extracellular polysaccharides, but sulphated ones have only been seen in cyanobacteria.

Maeda et al. showed that the sulphated polysaccharide in *Synechocystis*, which they named Synechan, helps the cyanobacterium to form buoyant aggregates by trapping oxygen bubbles in the slimy web of cells and polysaccharides. This suggests that a major purpose for the production of Synechan is buoyancy.

Previous studies on *Synechocystis* have shown that type IV pili, which decorate the surface of cyanobacteria, also play a role in forming blooms (Allen et al., 2019; Conradi et al., 2019). These retractable and adhesive protein fibres are important for motility, adhesion to substrates and DNA uptake (Schuergers and Wilde, 2015). The formation of blooms may require both type IV pili and Synechan – for example, the pili may help to export the polysaccharide outside the cell. Indeed, the activity of these protein fibres may be connected to the production of extracellular polysaccharides in filamentous cyanobacteria (Khayatan et al., 2015). A more obvious answer would be that pili help to build the aggregates by binding the cells with each other or with the extracellular polysaccharide. As with other kinds of bacteria (Adams et al., 2019), certain components of the pili may allow cyanobacteria from the same species to recognise each other and make initial contacts, which are then stabilised by building a mass of extracellular polysaccharide.

The ‘bubble flotation’ mechanism identified by Maeda et al. joins a range of known strategies that enable cyanobacteria to control their buoyancy, such as using gas vesicles or accumulating carbohydrate ballasts (Figure 1; Kromkamp and Walsby, 1990). Type IV pili on their own could also control the position of marine cyanobacteria in the water column by regulating viscous drag (Aguilo-Ferretjans et al., 2021). Extracellular polysaccharide appears to be a multipurpose asset for cyanobacteria, from floatation device to food storage, defence mechanism and mobility aid (Khayatan et al., 2015). Cyanobacteria can make surprisingly complex and diverse lifestyle choices, and the role of slime in their social life calls for further exploration.

**Figure 1.**
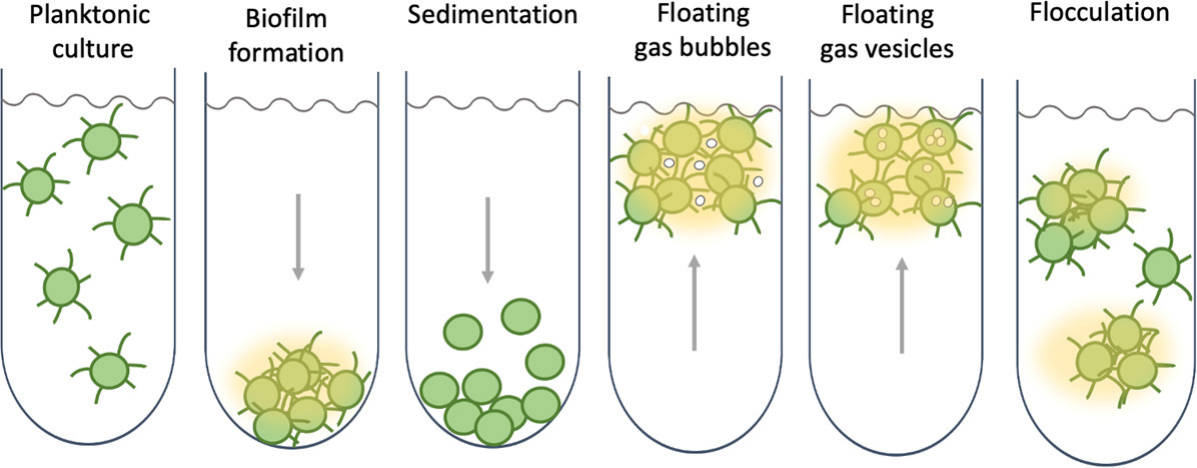
Collective behaviour and lifestyle choices in single-celled cyanobacteria. Bacteria can stay in suspension as individual cells, adhere collectively to surfaces to form biofilms, passively sediment, or flocculate to form suspended aggregates. Cyanobacteria are able to produce sulphated polysaccharides (yellow haze surrounding clumps of cells) that enable them to form floating aggregates. Maeda et al. discovered that the oxygen produced by the cyanobacteria becomes trapped in the network of polysaccharides and cells, enabling the microorganisms to form buoyant blooms. It is thought that specific protein fibres known as pili (represented as lines radiating from the cells) may act as an additional way to link cells to each other or onto surfaces. Some cyanobacteria also use sophisticated intracellular gas vesicles as floating aids.

It remains to be seen if Synechan production in nature would serve to segregate cyanobacteria away from other species, including dangerous predators; or whether it may help to build a floating microbial community where multiple, metabolically diverse species can cooperate. We know so little about the real life of *Synechocystis* outside the laboratory that both ideas are equally possible.
